# Impact of cumulative exposure to high-dose oral glucocorticoids on fracture risk in Denmark: a population-based case-control study

**DOI:** 10.1007/s11657-018-0424-x

**Published:** 2018-03-18

**Authors:** M. Amine Amiche, Shahab Abtahi, Johanna H. M. Driessen, Peter Vestergaard, Frank de Vries, Suzanne M. Cadarette, Andrea M. Burden

**Affiliations:** 1 Leslie Dan Faculty of Pharmacy, University of Toronto, Toronto, ON Canada; 2 Department of Clinical Pharmacy and Toxicology, Maastricht University Medical Centre+, Maastricht, The Netherlands; 3 Care and Public Health Research Institute (CAPHRI), Maastricht, The Netherlands; 4 Division of Pharmacoepidemiology and Clinical Pharmacology, Utrecht Institute of Pharmaceutical Sciences, Utrecht University, Utrecht, The Netherlands; 5 NUTRIM School for Nutrition and Translational Research in Metabolism, Maastricht University, Maastricht, The Netherlands; 6 Department of Clinical Medicine, Aalborg University, Aalborg, Denmark; 7 Department of Endocrinology, Aalborg University Hospital, Aalborg, Denmark

**Keywords:** Oral glucocorticoids, Osteoporosis, Hip fracture, Case-control

## Abstract

**Summary:**

We examined the effect of cumulative exposure to high doses of oral glucocorticoids on fracture risk. Compared to short-course users (daily dose ≥ 15 mg + cumulative < 1 g), heavy users (daily dose ≥ 15 mg + cumulative dose ≥ 1 g) had the highest risk of fracture. These patients should be monitored for fracture management strategies.

**Purpose:**

The effect of cumulative exposure to high daily doses of oral glucocorticoids on fracture risk remains debated. We therefore aimed to examine the hip fracture risk associated with short courses and heavy use of high-dosed oral glucocorticoids.

**Methods:**

We conducted a population-based case-control study using the Danish National Health Service data, 1996–2011. Cases were those aged ≥ 18 years who sustained a hip (primary outcome) fracture (*n* = 81,342). Vertebral and forearm fractures were considered in secondary analyses. Controls (matched 1:1) were those without a fracture. Average daily dose (DD) and total cumulative dose (CD) were calculated among current oral glucocorticoid users. Among patients with a high daily dose (DD ≥ 15 mg), we identified short-course users as those with a CD < 1 g and heavy users as those with a CD ≥ 1 g. We estimated adjusted odds ratio (adj.OR) of fracture with current glucocorticoid use compared to never-use, using conditional logistic regression.

**Results:**

A high DD (≥ 15 mg) and high CD (≥ 1 g) were independently associated with an increased hip fracture risk (adj.OR 2.5; 95% CI 2.2–2.9; adj.OR 1.6; 95% CI 1.5–1.8, respectively). However, the risk was substantially increased among heavy users (DD ≥ 15 mg and CD ≥ 1 g: adj.OR 2.9; 95% CI 2.5–3.4) as compared to short-course users (DD ≥ 15 mg and CD < 1 g: adj.OR 1.4; 95% CI 1.1–1.9). Associations were stronger for vertebral fractures, yet little association was identified for forearm fractures.

**Conclusion:**

Among patients receiving a high DD (≥ 15 mg), heavy users (≥ 1 g CD) showed the most substantial increase in hip fracture risk. Among those receiving high DD, a threshold of 1 g CD may identify heavy users that are candidates for focused fracture management services.

**Electronic supplementary material:**

The online version of this article (10.1007/s11657-018-0424-x) contains supplementary material, which is available to authorized users.

## Background

Oral glucocorticoids are widely prescribed drugs with established clinical benefits for patients with chronic inflammatory and autoimmune diseases, such as chronic respiratory disease, inflammatory arthritis, and dermatologic disease [1–3]. It is estimated that the prevalence of oral glucocorticoid use among adults ranges between 1.5 and 3% worldwide [4]. Unfortunately, oral glucocorticoid use is limited by significant side effects that usually appear after an extended period of exposure [5–7]. Glucocorticoid-induced musculoskeletal disorders, such as osteoporosis, are a major problem and a well-documented side effect [8–13]. Indeed, it is estimated that oral glucocorticoids are associated with a 30 to 120% increased risk of hip fracture and 2- to 3-fold increase in vertebral fracture risk compared with non-use [14–16]. For inflammatory conditions, short courses of high doses with tapering regimens are often required for symptom management. While it is well-known that oral glucocorticoid-induced bone loss and fracture risk is dose-dependent [15, 17], the relationship with the cumulative exposure is less well established [15, 17].

To minimize fracture risk, clinical practice guidelines recommend that osteoporosis pharmacotherapy should be given to patients that are expected to receive a daily dose of 5 to 7.5 mg of prednisone equivalent for 3–6 months [10, 18–20]. However, in a real-world setting, patients with inflammatory conditions often receive intermittent short courses (7–14 days) of high doses (40–60 mg per day), or a continuous low dose (5–10 mg per day) for longer periods until remission of the underlying disease [21–23]. In both cases, this may result in a similar cumulative exposure; however, the impact on bone and fracture may differ due to the daily dose.

To date, data related to the risk of bone fracture associated with these different patterns of exposure are limited, and it is often difficult to examine different cumulative and daily dose exposure patterns in database research. Thus, in this study using population-level data from Denmark, we sought to examine the association between the daily dose and cumulative exposure to oral glucocorticoids and the risk of fracture. Hip fracture is the most burdensome osteoporotic fracture, and its identification using hospital and physician diagnosis codes is accurate compared to other common osteoporosis fractures [24, 25]. In particular, we focus on the effect of “short courses” or “heavy use” of high daily doses of oral glucocorticoids and hip fracture risk. Additionally, as the association of oral glucocorticoid exposure with other fractures (forearm and vertebra) is not yet clear, we further examined other fracture sites in secondary analyses.

## Methods

### Data sources

We utilized data from the Danish National Health Service Register that covers all contacts with the health sector for over five million individuals in Denmark [26]. The National Health Service Register captures all contacts with general practitioners. The National Hospital Discharge Register includes information on hospital admissions since 1977 and all outpatient and emergency department visits since 1995 [27]. All diagnoses are coded using the International Classification of Diseases and Related Health Problems (ICD) system, with high precision for diagnoses, particularly for fractures [28]. The vital status for the entire Danish population is identified from the Civil Registration System. The Danish Medicines Agency Register of Medicinal Product Statistics is a nationwide prescription database that uses the Anatomical Therapeutic Chemical Classification (ATC) system and includes information on the type, amount, and prescription date. All registers can be linked at the patient level using the unique 10-digit civil registry number assigned to all Danish citizens [29].

### Study design

We completed a population-based case-control study. Cases were all patients aged 18 years or older, who sustained the first ever hip, vertebral, or forearm fracture between January 1, 1996, and December 31, 2011. The primary fracture of interest was hip fracture (ICD-10 codes: S72.0–S72.2). We further identified patients with clinical symptomatic vertebral fractures (ICD-10 codes: S12, S22.0, S22.1, S32.0, T08) and forearm fractures: radius or ulna (ICD-10 code: S52).

We randomly selected a control for each case, matched on age and year of birth, using incidence density sampling. Controls had no fracture during the study period. The date of the first fracture was used as the index date for the cases, and controls were assigned the index date of their matched case.

### Exposure

We defined oral glucocorticoid exposure based on most recent glucocorticoid prescription prior to the index date: current (within 91 days), recent (92–182 days), past (183–364 days), and distant past (≥ 365 days). Patients with no glucocorticoid prescriptions prior to the index date were classified as never users and were the referent category in all analyses.

**Fig. 1:**
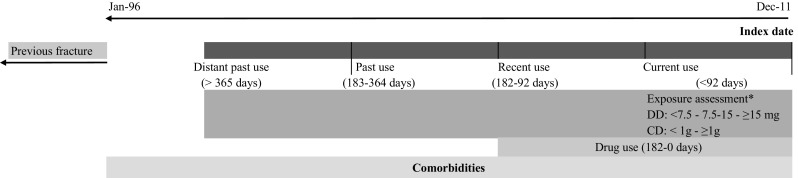
Study design diagram. *DD: average daily dose in milligrams of prednisone equivalent measured among current users. *CD: cumulative exposure in grams of prednisone equivalent measured among current users. Notes: index date: defined as the first fracture occurring between 1996 and 2011. Oral glucocorticoid exposure based on most recent glucocorticoid prescription prior to the index date: current (within 91 days), recent (92–182 days), past (183–364 days), and distant past (> 364 days). Comorbidities: any diagnoses code prior to the index date. Drug use: any drug prescription within 6 months prior to the index date. Previous fracture: any fracture prior to January 1996

Average daily dose (DD) and the cumulative dose (CD) of oral glucocorticoids were calculated among current users and expressed as prednisone equivalents. The average DD was calculated by dividing CD by the treatment time (days between the first glucocorticoid prescription to the index date), and categorized into three groups: low (< 7.5 mg), moderate (7.5–14.9 mg), and high (≥ 15 mg). CD was calculated by summing defined daily doses of glucocorticoid prescriptions prior to the index date, according to the World Health Organization. The primary exposure categories of interest were defined as follows: CD was categorized as low (< 1 g CD) or high (≥ 1 g CD) (Fig. 1). In a secondary analysis, we defined high CD as ≥ 5 or ≥ 10 g, with relevant sub-groups of CD (1.0–4.9 g, and 5.0–9.9 g), where appropriate.

To examine fracture risk associated with different patterns of glucocorticoid exposure, we further stratified exposure by both DD and CD to capture short-course and heavy users of high DD (≥ 15 mg) oral glucocorticoids. Among these patients with a high DD, we defined “short-course users” as those with < 1 g CD, while “heavy users” were those with a CD ≥ 1 g. These definitions were used as a heavy user would be a patient receiving 2–3 months of 15 mg daily, or more than a 30-day exposure to 40–60 mg daily.

### Covariates

A history of the following comorbidities were identified if they occurred any time before a patient’s index date: secondary osteoporosis (diabetes type 1, hypogonadism, or premature menopause), fracture (prior to 1996), rheumatoid arthritis, gout, inflammatory bowel disease, chronic obstructive pulmonary disease, alcoholism, cerebrovascular disease, congestive heart failure, pneumonia, type 2 diabetes mellitus, hyperthyroidism, hypothyroidism, malignancies (excluding non-melanoma skin cancer), dementia, and retinopathy. All potential confounders were identified using ICD-8 or ICD-10 codes. In the 6 months before the index date, we identified the following prescriptions as potential confounders: bone-sparing drugs (bisphosphonates, vitamin D, calcium, calcitonin, denosumab, raloxifene, and strontium ranelate), hormone replacement therapy, parathyroid hormone, antidepressants, antipsychotics, hypnotics/anxiolytics, anticonvulsants, anti-Parkinson drugs, inhaled bronchodilators, inhaled corticosteroids, xanthine derivatives, antihypertensive drugs, and proton pump inhibitors.

### Statistical analysis

Conditional logistic regression was used to estimate the association between the use of oral glucocorticoids and fracture risk. All results are presented as odds ratios (ORs) with the corresponding 95% confidence intervals (95% CIs). Analyses were stratified by DD, CD, and the DD stratified by CD (i.e., short-course vs. heavy use). Final regression models were determined using stepwise backward elimination using a significance level of 0.05. We completed an additional analysis that adjusted the final model for osteoporosis medications in addition to identified significant covariates. Separate models were run for hip (primary outcome), clinical symptomatic vertebral, and forearm fracture. Analyses were conducted using SAS software version 9.3 (SAS Institute Inc., Cary, NC).

## Results

### Primary fracture site: hip fracture

We identified 81,342 cases of hip fracture who were well matched to controls on age (mean 78.6 years, standard deviation 12 years) and sex (women 68.5%) (Table 1). Compared with controls, a higher proportion of cases had comorbidities, such as the history of fracture (27 cases vs. 11% controls), chronic obstructive pulmonary disease (10 cases vs. 7% controls), and secondary osteoporosis (8 cases vs. 5% controls). Drug use in the 6 months prior to the index date was higher among the fracture cases, as compared to controls: bisphosphonates (3.5 vs. 2.4%), antipsychotics (9.3 vs. 4.3%), anticonvulsants (4.5 vs. 1.9%), and antidepressants (22.3 vs. 10.8%) (Table 1).

**Table 1:** Baseline characteristics of cases of hip fracture and controls

	Cases (*n* = 81,342)		Controls (*n* = 81,342)		
Characteristics	*N*	%	*n*	%	*p* value
Women	55,776	68.6	55,776	68.6	1
Mean age in years (SD)	78.6	(12.0)	78.6	(12.0)	0.99
Age group					
18–49 years	2237	2.8	2240	2.8	0.99
50–59 years	3791	4.7	3794	4.7	0.99
60–69 years	8565	10.5	8578	10.6	0.91
70–79 years	21,513	26.5	21,545	26.5	0.86
80+ years	45,236	55.6	45,185	55.6	0.8
History of comorbidities					
Chronic obstructive pulmonary disease	7986	9.8	5293	6.5	< 0.001
Fracture (prior to 1996)	21,653	26.6	8672	10.7	< 0.001
Rheumatoid arthritis	2318	2.9	1452	1.8	< 0.001
Inflammatory bowel disease	1998	2.5	1405	1.7	< 0.001
Secondary osteoporosis^a^	6189	7.6	3951	4.9	< 0.001
Drug use 6 months before index date					
Bisphosphonates	2868	3.5	1917	2.4	< 0.001
Vitamin D	156	0.2	122	0.2	< 0.05
Calcium	1718	2.1	1109	1.4	< 0.001
Raloxifene	104	0.1	62	0.1	< 0.01
Strontium ranelate	37	0.1	19	0.0	0.01
Denosumab	8	0.0	–	–	–
Parathyroid hormone	30	0.0	20	0.0	0.16
Hormone replacement therapy	4306	5.3	5875	7.2	< 0.001
Inhaled corticosteroids	2948	3.6	2625	3.2	< 0.001
Inhaled bronchodilators	7020	8.6	5436	6.7	< 0.001
Antipsychotics	7552	9.3	3496	4.3	< 0.001
Antidepressants	18,138	22.3	8783	10.8	< 0.001
Hypnotics/anxiolytics	13,053	16.1	8734	10.7	< 0.001
Anticonvulsants	3660	4.5	1539	1.9	< 0.001
Anti-Parkinsonian drugs	2124	2.6	841	1.0	< 0.001

Comorbidities: any diagnoses code (ICD-8 or ICD-10) recorded prior to the index date. Drug use: any drug prescription within 6 months prior to the index date. Cells < 6 are not reported

*SD* standard deviation

^a^Secondary osteoporosis defined as a diagnosis of diabetes type 1, hypogonadism, or premature menopause

**Fig. 2:**
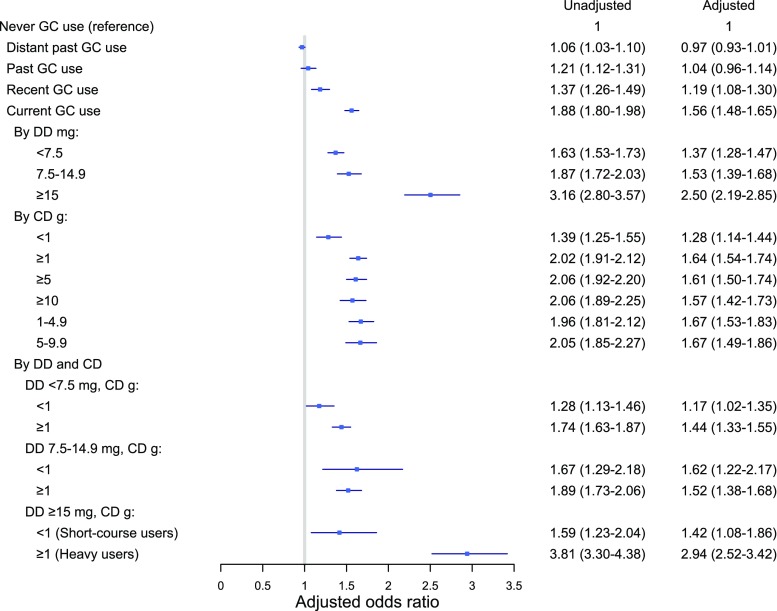
Odds ratio of hip fracture by glucocorticoid use (vs. never) [GC, glucocorticoid, DD (average daily dose), CD (cumulative dose)]. Oral glucocorticoid exposure based on most recent glucocorticoid prescription prior to the index date: current (within 91 days), recent (92–182 days), past (183–364 days), and distant past (> 364 days). DD and CD were calculated among current users. Adjusted for history of chronic obstructive pulmonary disease, fracture (prior to 1996), rheumatoid arthritis, inflammatory bowel disease, secondary osteoporosis, antidepressants, anxiolytics and hypnotics, anticonvulsants bone-sparing drugs, and inhaled bronchodilators

A total of 16,606 patients with hip fracture (20.4%) and 13,763 controls (17.0%) had used oral glucocorticoids prior to the index fracture (Supplementary material, Table 1). Current use of oral glucocorticoids was associated with an increased risk of hip fracture (adjusted [adj] OR 1.56, 95% CI [1.48–1.65]), as compared to never users of oral glucocorticoids (Fig. 2). Among current users, a dose-response relationship was observed with increasing DD: < 7.5 mg (adj.OR 1.37 [95% CI 1.23–1.47]), 7.5–14.9 mg (adj.OR 1.53 [95% CI 1.39–1.68]), and ≥ 15 mg (adj.OR 2.5 [95% CI 2.19–2.85]) (Appendix Table 1a). Likewise, a higher CD was associated with an increased hip fracture risk. A CD < 1 g was associated with a 30% increased hip fracture risk (adj.OR 1.28 [95% CI 1.14–1.44]), while a 60% increased risk was observed for CD ≥ 1 g (adj.OR 1.64 [95% CI 1.54–1.74]), CD ≥ 5 g (adj. OR 1.61 [95% CI 1.50–1.74]), and CD ≥ 10 g (adj.OR 1.57 [95% CI 1.42–1.73]).

To examine the association between “heavy” and “short-course” users of high-dosed oral glucocorticoids, the DD was further stratified by the CD (Fig. 2). Among short-course users (DD ≥ 15 mg and CD < 1 g), a 42% increase in hip fracture risk was observed (adj.OR 1.42 [95% CI 1.08–1.86]). In contrast, “heavy” use of high doses (DD ≥ 15 mg and CD ≥ 1 g) resulted in a tripled risk of hip fracture (adj.OR 2.94 [95% CI 2.52–3.42]). When CD exceeded 5 or 10 g, among those with a high DD, the hip fracture risk was similar (CD ≥ 5 g: adj.OR 2.86 [95% CI 2.29–3.58] and CD ≥ 10 g: adj.OR 2.55 [95% CI 1.84–3.55]).

### Secondary fracture sites: vertebral and forearm fractures

**Fig. 3:**
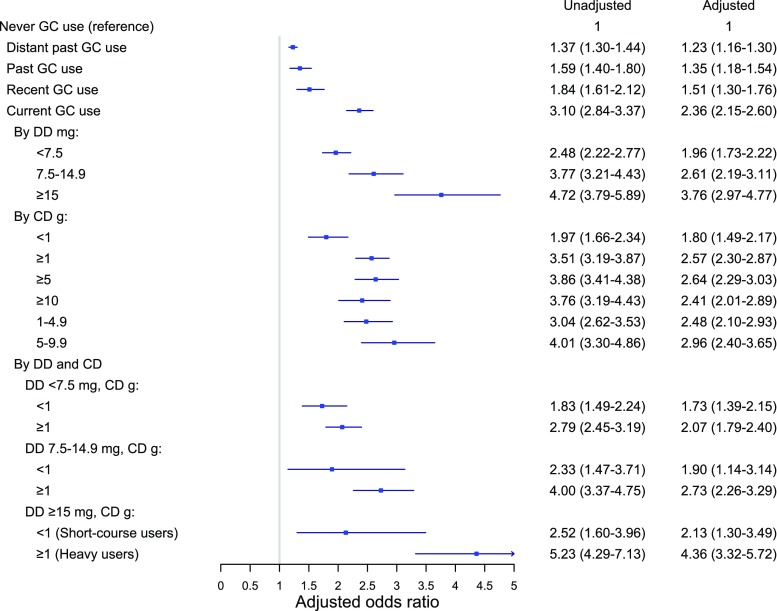
Odds ratio of vertebral fracture by glucocorticoid dose (vs. never) [GC, glucocorticoid, DD (average daily dose), CD (cumulative dose)]. Oral glucocorticoid exposure based on most recent glucocorticoid prescription prior to the index date: current (within 91 days), recent (92–182 days), past (183–364 days), and distant past (> 364 days). DD and CD were calculated among current users. Adjusted for history of chronic obstructive pulmonary disease, fracture (prior to 1996), rheumatoid arthritis, inflammatory bowel disease, secondary osteoporosis, antidepressants, anxiolytics and hypnotics, anticonvulsants bone-sparing drugs, and inhaled bronchodilators

Figure 3 and Appendix Table 2 present the ORs of clinical symptomatic vertebral fracture by glucocorticoid exposure. Current use of oral glucocorticoids was associated with doubled risk of clinical symptomatic fracture (adj. OR 2.36 [95% CI 2.15–2.60]). A high DD (≥ 15 mg/day) was associated with a 3.8-fold increased clinical vertebral fracture risk (adj.OR 3.76 [95% CI 2.97–4.77]), and a CD ≥ 1 g was associated with a tripled risk (adj.OR 2.96 [95% CI 2.40–3.65]). Among the high DD users, short-course use was associated with a doubled risk of clinical symptomatic vertebral fracture risk (adj.OR 2.13, 95% CI 1.30–3.49), while “heavy” use was associated with a more than 4-fold increased risk (adj.OR 4.36, 95% CI 3.32–5.72) (Fig. 3 and Appendix Table 2).

**Fig. 4:**
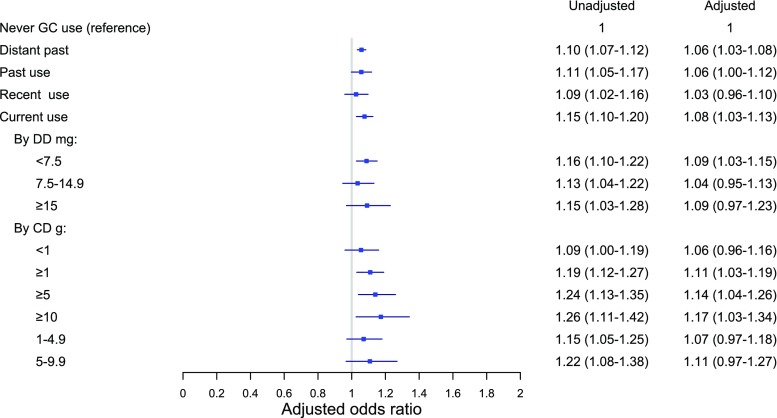
Odds ratio of forearm fracture by glucocorticoid use (vs. never) [GC, glucocorticoid, DD (average daily dose), CD (cumulative dose)]. Oral glucocorticoid exposure based on most recent glucocorticoid prescription prior to the index date: current (within 91 days), recent (92–182 days), past (183–364 days), and distant past (> 364 days). DD and CD were calculated among current users. Adjusted for history of chronic obstructive pulmonary disease, fracture (prior to 1996), rheumatoid arthritis, inflammatory bowel disease, secondary osteoporosis, antidepressants, anxiolytics and hypnotics, anticonvulsants

Among patients with forearm fractures, the analysis showed minimal to no association with oral glucocorticoid exposure (Fig. 4). No dose-response of forearm fracture risk was observed (Fig. 4 and Appendix Table 3).

## Discussion

In this population-based case-control study, we identified that heavy use (high DD and high CD) of oral glucocorticoids was associated with a 3-fold increased hip fracture risk, which was substantially higher as compared to short-course users. While both the DD and CD were independently associated with hip fracture risk, our results suggest that hip fracture risk was modified by the CD among patients receiving a high DD of oral glucocorticoids. This association was not observed among patients with a low to moderate DD, thereby suggesting that heavy users of high DD oral glucocorticoids are a distinct patient group with substantially elevated fracture risk.

Interestingly, in our study, we did not observe an incremental increase in hip fracture risk among patients receiving CD exceeding 1 g of prednisone equivalent (≥ 5 or ≥ 10 g). This result may indicate a threshold effect for the CD, which may be used to guide clinical decision making to determine patients in need of fracture/osteoporosis management. Our study further showed that the odds of sustaining fractures among glucocorticoid users were stronger for clinical symptomatic vertebral fracture than hip fracture, while forearm fracture risk was minimal to non-significant among current users and across dose categories.

Systemic glucocorticoids cause considerable inhibition of bone formation and bone resorption, but particularly when given at higher doses and longer durations. Additionally, systemic glucocorticoids impair renal and intestinal calcium absorption, reduce sexual hormone secretion, and cause muscle atrophy and gait impairment, which in turn increase bone loss and fracture risk [5]. These pleiotropic effects significantly increase fracture risk, and we speculate that it is the high daily and cumulative doses which can explain the effect modification that we observed. Our finding is also in line with a previous cohort study in the UK [15].

In clinical practice, treatment with bisphosphonates for osteoporosis management is often suboptimal among patients receiving oral glucocorticoids, particularly those receiving short courses as they do not reach the guideline recommendation of 7.5 mg per day for a duration of 3 months. As a result, these patients remain at high risk for potentially devastating hip fractures. Since the cycle of bone remodeling takes on average 3 months [30], multiple short courses are likely to hinder bone metabolism by preventing the skeleton to completely regenerate. Indeed, data have shown that fracture risk can persist up to 1 year after oral glucocorticoid cessation [15, 17]. However, the association between a more intermittent exposure to high DD and fracture remains controversial. Moreover, the effect of a CD of oral glucocorticoids, particularly in relation to the DD, on fracture risk remains debated.

While it is well established that a DD exceeding 15 mg is associated with a significant increase in fracture risk, our study highlighted that the magnitude of fracture risk associated with a high DD differed depending on the CD thresholds. We observed that patients receiving short courses (< 1 g CD) of high DD had an elevated hip and clinical symptomatic vertebral fracture risk, yet heavy users (≥ 1 g CD) of high DD had a tripled risk of hip fracture, and a 4.5-fold increase in clinical symptomatic vertebral fracture risk. Interestingly, we observed that fracture risk reached a plateau starting at a CD ≥ 1 g. Prior studies have shown that glucocorticoid daily dose is a stronger predictor than the cumulative dose [12, 15, 16]. However, our results suggest that high DD with low CD does not confer higher risk of fractures, and therefore, we assume that there is a threshold duration of exposure or a minimum number of high daily doses that are causing the highest risk of fracture. Our results support that a clinical threshold of 1 g prednisone equivalent may be useful to guide therapeutic interventions to prevent osteoporotic fracture. Indeed, clinical practice guidelines use various oral glucocorticoid exposure thresholds to consider anti-fracture treatment, yet no agreement between guidelines is established [10, 18–20].

When placing our results in the context of the literature, we note that the most comparable study to ours is a large cohort study conducted by de Vries et al., using the UK primary care data (Clinical Practice Research Datalink) to examine fracture risk among glucocorticoid users covering the period between 1987 and 1997 [15]. In their study, de Vries et al. examined similar glucocorticoid DD and CD exposure to our study, and showed a 49% increase in relative risk of hip and femur fracture, and more than 3-fold risk increase of vertebral fracture. The authors showed similar dose-response patterns for the average DD and CD and osteoporotic fracture overall. However, contrary to our study, de Vries et al. assessed fracture risk by disease at baseline, limiting the direct comparison with our study. Additionally, the comparator group in the study by de Vries et al. were those with past use of glucocorticoids, while never users were compared in our study. Thus, a larger overall effect of current use in our study would be expected.

Additionally, a case-control study in the Netherlands investigated inhaled and oral glucocorticoid users from 1991 to 2002, identifying cases of 366 hip or femur fracture among current oral glucocorticoid users [31]. This study examined daily dose-response and showed lower overall risk than identified in our study. However, this study did not consider the total CD of glucocorticoids, which is an important factor to estimate differential fracture risk. Consequently, we are unable to make a direct comparison regarding the effect of heavy or short-term use on fracture risk. Finally, Vestergaard et al. showed that the highest increase in hip fracture risk by cumulative dose was among patients receiving ≥ 1500 mg prednisone equivalent in the year preceding the fracture (OR 4.2 95% CI [1.96–9.00] vs. OR 1.89[1.62–2.21] for patients receiving 500–1499 mg prednisone equivalent) [32]. However, this analysis was not stratified by glucocorticoid daily dose.

Other observational studies have examined the association of short courses of glucocorticoid exposure and support our findings of a moderately increased fracture risk. A cohort study using administrative healthcare databases reported a similar increase in hip fracture risk (60%) among all glucocorticoid users, and RR of hip fracture equal to 1.26 (95% CI 0.87–1.83) among patients with short courses of oral glucocorticoids (< 90 days). A lack of statistical power is likely to cause the non-significant results for short courses. Another cohort study reported increased risk of hip fracture among oral glucocorticoid users (RR 1.87, 95% CI 1.19–2.94) [16]. In addition, increase in daily dose and duration were significantly associated with hip and clinical symptomatic vertebral fracture risk. This study showed that short duration of oral glucocorticoid exposure also increased fracture risk.

In interpreting our data, we are mindful of some limitations. By design, the case-control study is not able to provide an absolute risk. We used a density sampling technique which allowed us to interpret the odds ratio as risk ratio since controls were selected based on the person-time duration corresponding to each case [33]. There remains debate of the thresholds of glucocorticoid exposure resulting in fracture risk. Consequently, our definition of dose and duration thresholds may be debatable. Many guidelines recommend treatment among patients receiving a planned systemic glucocorticoid dose ≥ 7.5 mg prednisone equivalent daily and a duration of 3 months [19, 34]. However, the patterns of glucocorticoid exposure are often complex, requiring multiple courses and may therefore not meet guidelines for treatment, leaving many patients at risk. While the definition of high glucocorticoid daily doses is debated, doses < 7.5 mg/day prednisone equivalent are often considered as low doses in clinical trials of oral glucocorticoids [35, 36]. While our study did not identify significant differentiation between those receiving < 7.5 mg daily and those receiving 7.5–14.9 mg daily, there was a significant increased hip and vertebral fracture risk among those receiving ≥ 15 mg daily.

Additionally, there may be limitations regarding our definition of short-course and heavy users of high DD oral glucocorticoids. In this study, we assumed patients with a DD ≥ 15 mg and CD < 1 g may have received intermittent use (short-courses) of oral glucocorticoid therapy, as this would suggest that a patient received two or fewer 30-day courses of 15 mg per day. Conversely, the heavy users with ≥ 1 g CD were more likely to have received more prolonged exposure to oral glucocorticoid therapy. Using a combination of CD and DD to describe patterns of exposure may not be the most optimal choice. In many clinical cases, dosing schedule of oral glucocorticoids consists of a high DD and a short duration. In this study, we assumed that less than 1 g of prednisone equivalent before the fracture event is indicative of short-course or intermittent use for acute symptom management. This would correspond to one or two 30-day courses of 15 mg per day. However, it is possible that intermittent users may accumulate more than 1 g of exposure if a DD exceeded 30 mg. Nonetheless, we expect that this is likely to happen in very few cases only.

Additionally, while we adjusted for common glucocorticoid indications, we did not stratify our results by the specific disease indications. Similarly, while we adjusted comorbidities and drug use, residual confounding remains a limitation. We recognize that other risk factors have not been captured in our data and therefore not adjusted for. These include patient body mass index, smoking status, and bone mineral density values. However, we note that de Vries et al. reported similar results using UK data, which adjusted for these variables, and there is evidence showing that the fracture risk could be partly independent from the diminished bone density in patients who take oral glucocorticoids [37].

Another limitation was the possibility of misclassification bias in the Danish database. Vertebral fractures can be asymptomatic and likely have a low predictive value when defined using claims data [38]. This may have resulted in an underestimated effect in our vertebral fracture results. However, we believe there is little chance of differential misclassification and commonly used codes for osteoporotic fracture were identified. Additionally, misclassification of exposure is possible with glucocorticoids as information was derived from pharmacy claims, and we cannot be assured that the pills were taken as prescribed. Indeed, glucocorticoids may be prescribed on a “take-as-needed” basis, and therefore may have been collected at the pharmacy and not taken. In this study, the average daily dose was used, yet we acknowledge that this method has limitations with oral glucocorticoids, particularly when the glucocorticoid regimen requires adjustment and gradual tapering for long exposure periods [39]. This may result in underestimating the DD. However, the CD may partially adjust for this by estimating the overall exposure.

There are several strengths to our current study. We used the Danish nationwide population register, which is a large population-based register that permitted the examination of a large number of cases and controls. In Denmark, all residents receive universal medical and prescription services. Thus, the data are complete and collected longitudinally. This data also permitted the inclusion of many important confounders. Additionally, the prescription data in Denmark are claims-based and collected longitudinally, permitting us to calculate a reliable cumulative and average daily dose. Moreover, all data on fractures have been validated [40], minimizing the effect of misclassification.

### Clinical implication

The literature is consistent with respect to increases in fracture risk related to oral glucocorticoid exposure, yet the independent and combined effects of DD and CD on fracture risk remains controversial. According to the underlying condition, patients are faced with various instructions regarding glucocorticoid exposure—including tapering over time, alternate-day use, and “take as needed” instructions. Thus, defining an exposure threshold to initiate osteoporosis treatment is ambiguous, and difficult to assess in research using population-based data. In general, the literature shows suboptimal osteoporosis management among oral glucocorticoid users, where up to 70% of eligible users have no bone protection treatment [3, 41]. Many oral glucocorticoid users may not fall under clinical guidelines’ eligibility criteria to receive osteoporosis management.

The results of this study, therefore, provide additional evidence that a high DD (≥ 15 mg/day) and a CD exceeding 1 g is likely to cause the greatest risk of hip and clinical symptomatic vertebral fracture. Thus, while patients receiving short courses of high DD should continue to be monitored, focused attention for fracture management and osteoporosis pharmacotherapy should be given to patients with heavy use. These results may help clinical guidance to elaborate an evidence-based strategy for bone protection among oral glucocorticoid users.

## Conclusion

In conclusion, heavy use of high-dosed oral glucocorticoids is associated with a substantial 3-fold increase in hip fracture risk. Therefore, while short courses or more intermittent use of high-dosed oral glucocorticoids do increase fracture risk, primary attention should be paid to patients with heavy use—defined as those receiving multiple short or prolonged courses of high glucocorticoid doses that result in a cumulative exposure exceeding 1 g prednisone equivalent. Knowing a patients’ prescription history to identify the cumulative exposure to high daily doses of oral glucocorticoids may help clinicians to identify patients that are at high risk of fractures. These patients should then be targeted for osteoporosis management strategies to minimize fracture risk.

## Electronic supplementary material

ESM 1 (DOCX 52 kb)

## Abbreviations

CD: Cumulative dose
CI: Confidence interval
DD: Daily dose
OR: Odds ratio
