# Supplementary material for: Impact of cumulative exposure to high-dose oral glucocorticoids on fracture risk in Denmark: a population-based case-control study
Source: Arch Osteoporos. 2018 Mar 18;13(1):30. doi: 10.1007/s11657-018-0424-x (PMC5857556; doi:10.1007/s11657-018-0424-x)
Supplement: Supplementary file 1 — (DOCX 52 kb) [file 11657_2018_424_MOESM1_ESM.docx]

**Supplementary Material**

Appendix Table 1. Results for hip fracture

|  | **No of cases**  **n=81,342** | **No of controls n=81,342** | **Unadjusted OR** | **95% CI** | | **Adjusted OR (a)** | **95% CI** | | **Adjusted OR (b)** | **95% CI** | |
| --- | --- | --- | --- | --- | --- | --- | --- | --- | --- | --- | --- |
| Never GC use | 64,736 | 67,579 | 1.00 | Reference | | 1.00 | Reference | | 1.00 | Reference | |
| Ever GC use | 16,606 | 13,763 | 1.28 | 1.25 | 1.32 | 1.12 | 1.08 | 1.15 | 1.11 | 1.08 | 1.15 |
| Distant past GC use | 8,639 | 8,610 | 1.06 | 1.03 | 1.10 | 0.96 | 0.93 | 1.00 | 0.97 | 0.93 | 1.01 |
| Past GC use | 1,407 | 1,213 | 1.21 | 1.12 | 1.31 | 1.04 | 0.96 | 1.14 | 1.04 | 0.96 | 1.14 |
| Recent GC use | 1,330 | 1,023 | 1.37 | 1.26 | 1.49 | 1.19 | 1.09 | 1.30 | 1.19 | 1.08 | 1.30 |
| **Current GC use** | 5,230 | 2,917 | 1.88 | 1.80 | 1.98 | 1.59 | 1.50 | 1.67 | 1.56 | 1.48 | 1.65 |
| **By average daily dose (oral prednisolone equivalents)** | | | | | | | | | | | |
| <7.5 mg/day | 2,628 | 1,699 | 1.63 | 1.53 | 1.73 | 1.39 | 1.29 | 1.49 | 1.37 | 1.28 | 1.47 |
| 7.5 -14.9 mg/day | 1,548 | 869 | 1.87 | 1.72 | 2.03 | 1.56 | 1.42 | 1.72 | 1.53 | 1.39 | 1.68 |
| ≥15 mg/day | 1,054 | 349 | 3.16 | 2.80 | 3.57 | 2.54 | 2.23 | 2.90 | 2.50 | 2.19 | 2.85 |
| **By cumulative dose (oral prednisolone equivalents)** | | | | | | | | | | | |
| <1gram | 812 | 608 | 1.39 | 1.25 | 1.55 | 1.28 | 1.14 | 1.43 | 1.28 | 1.14 | 1.44 |
| ≥1grams | 4,418 | 2,309 | 2.02 | 1.91 | 2.12 | 1.67 | 1.58 | 1.77 | 1.64 | 1.54 | 1.74 |
| ≥5grams | 2,666 | 1,370 | 2.06 | 1.92 | 2.20 | 1.66 | 1.54 | 1.79 | 1.61 | 1.50 | 1.74 |
| ≥ 10grams | 1,519 | 780 | 2.06 | 1.89 | 2.25 | 1.62 | 1.47 | 1.79 | 1.57 | 1.42 | 1.73 |
| 1-4.9grams | 1,752 | 939 | 1.96 | 1.81 | 2.12 | 1.69 | 1.55 | 1.84 | 1.67 | 1.53 | 1.83 |
| 5-9.9grams | 1,147 | 590 | 2.05 | 1.85 | 2.27 | 1.70 | 1.53 | 1.90 | 1.67 | 1.49 | 1.86 |
| **By average daily dose & cumulative dose** | | | | | | | | | | | |
| DD <7.5 mg/day | | | | | | | | | | | |
| CD < 1gram | 513 | 418 | 1.28 | 1.13 | 1.46 | 1.17 | 1.02 | 1.35 | 1.17 | 1.02 | 1.35 |
| CD ≥1 grams | 2,115 | 1,281 | 1.74 | 1.63 | 1.87 | 1.46 | 1.35 | 1.58 | 1.44 | 1.33 | 1.55 |
| CD ≥5 grams | 1,233 | 731 | 1.78 | 1.62 | 1.95 | 1.46 | 1.32 | 1.62 | 1.42 | 1.28 | 1.58 |
| CD ≥10 grams | 648 | 400 | 1.71 | 1.51 | 1.95 | 1.39 | 1.21 | 1.60 | 1.35 | 1.18 | 1.55 |
| CD 1-4.9grams | 882 | 550 | 1.70 | 1.52 | 1.89 | 1.46 | 1.30 | 1.64 | 1.46 | 1.29 | 1.64 |
| CD 5-9.9 grams | 585 | 331 | 1.86 | 1.63 | 2.14 | 1.55 | 1.33 | 1.79 | 1.51 | 1.30 | 1.75 |
| DD 7.5-14.9 mg/day | | | | |  |  |  |  |  |  |  |
| CD < 1 gram | 145 | 90 | 1.67 | 1.29 | 2.18 | 1.61 | 1.20 | 2.14 | 1.62 | 1.22 | 2.17 |
| CD ≥1 grams | 1,403 | 779 | 1.89 | 1.73 | 2.06 | 1.56 | 1.41 | 1.72 | 1.52 | 3.38 | 1.68 |
| CD ≥5 grams | 997 | 526 | 2.00 | 1.79 | 2.22 | 1.62 | 1.44 | 1.82 | 1.58 | 1.40 | 1.77 |
| CD ≥10 grams | 673 | 327 | 2.18 | 1.91 | 2.49 | 1.71 | 1.48 | 1.97 | 1.65 | 1.42 | 1.91 |
| CD 1-4.9grams | 406 | 253 | 1.67 | 1.42 | 1.95 | 1.43 | 1.21 | 1.70 | 1.40 | 1.18 | 1.67 |
| CD 5-9.9 grams | 324 | 199 | 1.70 | 1.42 | 2.03 | 1.48 | 1.22 | 1.80 | 1.45 | 1.19 | 1.76 |
| DD ≥15 mg/day | | | | | | | | | | | |
| CD < 1 gram | 154 | 100 | 1.59 | 1.23 | 2.04 | 1.41 | 1.07 | 1.84 | 1.42 | 1.08 | 1.86 |
| CD ≥1 grams | 900 | 249 | 3.81 | 3.30 | 4.38 | 3.00 | 2.58 | 3.50 | 2.94 | 2.52 | 3.42 |
| CD ≥5 grams | 436 | 113 | 4.09 | 3.32 | 5.03 | 2.95 | 2.35 | 3.68 | 2.86 | 2.29 | 3.58 |
| CD ≥10 grams | 198 | 53 | 3.93 | 2.90 | 5.33 | 2.66 | 1.92 | 3.70 | 2.55 | 1.84 | 3.55 |
| CD 1-4.9grams | 464 | 136 | 3.58 | 2.95 | 4.33 | 3.06 | 2.49 | 3.75 | 3.01 | 2.45 | 3.69 |
| CD 5-9.9 grams | 238 | 60 | 4.23 | 3.18 | 5.64 | 3.20 | 2.36 | 4.35 | 3.15 | 2.32 | 4.28 |
| (a) adjusted for: history of COPD, fracture, rheumatoid arthritis, inflammatory bowel disease, secondary osteoporosis, inhaled bronchodilators, antidepressants, anxiolytics and hypnotics, anticonvulsants | | | | | | | | | | | |
| (b) adjusted for confounders in model A plus bone medications (bisphosphonates, raloxifene, strontium, denosumab, calcium, vitamin D), calcitonin, and hormone replacement therapy | | | | | | | | | | | |
| Oral glucocorticoids exposure based on most recent glucocorticoid prescription prior to the index date: current (within 91-days), recent (92-182 days), past (183-364 days), and distant past (> 364 days). DD and CD were calculated among current users.  GC, Glucocorticoid; CD, Cumulative dose; DD, daily dose; OR, Odds ratio; CI, Confidence interval | | | | | | | | | | | |

Appendix Table 2. Results for clinical symptomatic vertebral fracture

|  | **No of cases**  **n=37,144** | **No of controls n=37,144** | **Unadjusted OR** | **95% CI** | | **Adjusted OR (a)** | **95% CI** | | **Adjusted OR (b)** | **95% CI** | |
| --- | --- | --- | --- | --- | --- | --- | --- | --- | --- | --- | --- |
| Never GC use | 30,044 | 32,463 | 1.00 | Reference | | 1.00 | Reference | | 1.00 | Reference | |
| Ever GC use | 7,100 | 4,681 | 1.72 | 1.65 | 1.79 | 1.49 | 1.42 | 1.56 | 1.44 | 1.38 | 1.52 |
| Distant past GC use | 3,745 | 3,103 | 1.37 | 1.30 | 1.44 | 1.24 | 1.17 | 1.32 | 1.23 | 1.16 | 1.30 |
| Past GC use | 637 | 450 | 1.59 | 1.40 | 1.80 | 1.38 | 1.20 | 1.57 | 1.35 | 1.18 | 1.54 |
| Recent GC use | 555 | 338 | 1.84 | 1.61 | 2.12 | 1.61 | 1.38 | 1.87 | 1.51 | 1.30 | 1.76 |
| **Current GC use** | 2,163 | 790 | 3.10 | 2.84 | 3.37 | 2.54 | 2.31 | 2.79 | 2.36 | 2.15 | 2.60 |
| **By average daily dose (oral prednisolone equivalents)** | | | | | | | | | | | |
| <7.5 mg/day | 1,060 | 485 | 2.48 | 2.22 | 2.77 | 2.07 | 1.84 | 2.34 | 1.96 | 1.73 | 2.22 |
| 7.5 -14.9 mg/day | 673 | 201 | 3.77 | 3.21 | 4.43 | 2.89 | 2.43 | 3.44 | 2.61 | 2.19 | 3.11 |
| ≥15 mg/day | 430 | 104 | 4.72 | 3.79 | 5.89 | 4.01 | 3.17 | 5.07 | 3.76 | 2.97 | 4.77 |
| **By cumulative dose (oral prednisolone equivalents)** | | | | | | | | | | | |
| <1gram | 376 | 212 | 1.97 | 1.66 | 2.34 | 1.81 | 1.50 | 2.18 | 1.80 | 1.49 | 2.17 |
| ≥1grams | 1,034 | 459 | 3.51 | 3.19 | 3.87 | 2.81 | 2.52 | 3.13 | 2.57 | 2.30 | 2.87 |
| ≥5grams | 1,129 | 331 | 3.86 | 3.41 | 4.38 | 2.98 | 2.60 | 3.42 | 2.64 | 2.29 | 3.03 |
| ≥ 10grams | 641 | 192 | 3.76 | 3.19 | 4.43 | 2.80 | 2.34 | 3.35 | 2.41 | 2.01 | 2.89 |
| 1-4.9grams | 658 | 247 | 3.04 | 2.62 | 3.53 | 2.57 | 2.18 | 3.03 | 2.48 | 2.10 | 2.93 |
| 5-9.9grams | 488 | 139 | 4.01 | 3.30 | 4.86 | 3.23 | 2.63 | 3.98 | 2.96 | 2.40 | 3.65 |
| **By average daily dose & cumulative dose** | | | | | | | | | | | |
| DD <7.5 mg/day | | | | | | | | | | | |
| CD < 1gram | 257 | 156 | 1.83 | 1.49 | 2.24 | 1.74 | 1.40 | 2.16 | 1.73 | 1.39 | 2.15 |
| CD ≥1 grams | 803 | 329 | 2.79 | 2.45 | 3.19 | 2.24 | 1.93 | 2.59 | 2.07 | 1.79 | 2.40 |
| CD ≥5 grams | 462 | 174 | 3.04 | 2.55 | 3.63 | 2.43 | 2.01 | 2.95 | 2.16 | 1.78 | 2.63 |
| CD ≥10 grams | 234 | 98 | 2.73 | 2.15 | 3.48 | 2.21 | 1.70 | 2.88 | 1.88 | 1.44 | 2.46 |
| CD 1-4.9grams | 341 | 155 | 2.51 | 2.07 | 3.05 | 2.02 | 1.63 | 2.49 | 1.97 | 1.59 | 2.44 |
| CD 5-9.9 grams | 228 | 76 | 3.43 | 2.64 | 4.45 | 2.71 | 2.04 | 3.59 | 2.51 | 1.89 | 3.34 |
| DD 7.5-14.9 mg/day | | | | | | | | | | | |
| CD < 1 gram | 56 | 27 | 2.33 | 1.47 | 3.71 | 1.90 | 1.15 | 3.15 | 1.90 | 1.14 | 3.14 |
| CD ≥1 grams | 617 | 174 | 4.00 | 3.37 | 4.75 | 3.06 | 2.54 | 3.68 | 2.73 | 2.26 | 3.29 |
| CD ≥5 grams | 451 | 117 | 4.33 | 3.53 | 5.33 | 3.19 | 2.56 | 3.99 | 2.78 | 2.23 | 3.48 |
| CD ≥10 grams | 295 | 73 | 4.55 | 3.51 | 5.88 | 3.18 | 2.42 | 4.19 | 2.75 | 2.09 | 3.63 |
| CD 1-4.9grams | 166 | 57 | 3.31 | 2.44 | 4.50 | 2.76 | 1.98 | 3.86 | 2.60 | 1.86 | 3.64 |
| CD 5-9.9 grams | 156 | 44 | 3.98 | 2.82 | 5.60 | 3.21 | 2.23 | 4.61 | 2.84 | 1.97 | 4.11 |
| DD ≥15 mg/day | | | | | | | | | | | |
| CD < 1 gram | 63 | 29 | 2.52 | 1.60 | 3.96 | 2.17 | 1.33 | 3.55 | 2.13 | 1.30 | 3.49 |
| CD ≥1 grams | 367 | 75 | 5.23 | 4.29 | 7.13 | 4.69 | 3.59 | 6.14 | 4.36 | 3.32 | 5.72 |
| CD ≥5 grams | 216 | 40 | 6.11 | 4.32 | 8.65 | 4.64 | 3.22 | 6.69 | 4.18 | 2.88 | 6.04 |
| CD ≥10 grams | 112 | 21 | 5.72 | 3.58 | 9.14 | 3.96 | 2.42 | 6.49 | 3.41 | 2.07 | 5.62 |
| CD 1-4.9grams | 151 | 35 | 4.88 | 3.35 | 7.09 | 4.76 | 3.20 | 7.08 | 4.58 | 3.08 | 6.83 |
| CD 5-9.9 grams | 104 | 19 | 6.60 | 3.94 | 11.07 | 5.52 | 3.22 | 9.47 | 5.19 | 2.99 | 9.00 |
| (a) adjusted for: history of COPD, fracture, rheumatoid arthritis, inflammatory bowel disease, secondary osteoporosis, inhaled bronchodilators, antidepressants, anxiolytics and hypnotics, anticonvulsants | | | | | | | | | | | |
| (b) adjusted for confounders in model A plus bone medications (bisphosphonates, raloxifene, strontium, denosumab, calcium, vitamin D), calcitonin, and hormone replacement therapy | | | | | | | | | | | |
| Oral glucocorticoids exposure based on most recent glucocorticoid prescription prior to the index date: current (within 91-days), recent (92-182 days), past (183-364 days), and distant past (> 364 days). DD and CD were calculated among current users.  GC, Glucocorticoid; CD, Cumulative dose; DD, daily dose; OR, Odds ratio; CI, Confidence interval | | | | | | | | | | | |

Appendix Table 3. Results for radius/ulna fracture

|  | **No of cases**  **n=201,963** | **No of controls n=201,963** | **Unadjusted OR** | **95% CI** | | **Adjusted OR (a)** | **95% CI** | | **Adjusted OR (b)** | **95% CI** | |
| --- | --- | --- | --- | --- | --- | --- | --- | --- | --- | --- | --- |
| Never GC use | 172,692 | 174,940 | 1.00 | Reference | | 1.00 | Reference | | 1.00 | Reference | |
| Ever GC use | 29,271 | 27,023 | 1.11 | 1.08 | 1.13 | 1.06 | 1.03 | 1.08 | 1.06 | 1.04 | 1.08 |
| Distant past GC use | 19,160 | 17,862 | 1.10 | 1.07 | 1.12 | 1.05 | 1.03 | 1.08 | 1.06 | 1.03 | 1.08 |
| Past GC use | 2,961 | 2,719 | 1.11 | 1.05 | 1.17 | 1.05 | 0.99 | 1.11 | 1.06 | 1.00 | 1.12 |
| Recent GC use | 2,085 | 1,949 | 1.09 | 1.02 | 1.16 | 1.03 | 0.96 | 1.10 | 1.03 | 0.96 | 1.10 |
| **Current GC use** | 5,065 | 4,493 | 1.15 | 1.10 | 1.20 | 1.09 | 1.05 | 1.14 | 1.08 | 1.03 | 1.13 |
| **By average daily dose (oral prednisolone equivalents)** | | | | | | | | | | | |
| <7.5 mg/day | 3,161 | 2,785 | 1.16 | 1.10 | 1.22 | 1.10 | 1.04 | 1.16 | 1.09 | 1.03 | 1.15 |
| 7.5 -14.9 mg/day | 1,229 | 1,111 | 1.13 | 1.04 | 1.22 | 1.08 | 0.99 | 1.18 | 1.04 | 0.95 | 1.13 |
| ≥15 mg/day | 675 | 597 | 1.15 | 1.03 | 1.28 | 1.10 | 0.98 | 1.24 | 1.09 | 0.97 | 1.23 |
| **By cumulative dose (oral prednisolone equivalents)** | | | | | | | | | | | |
| <1gram | 1,440 | 1,356 | 1.08 | 1.00 | 1.16 | 1.03 | 0.96 | 1.12 | 1.04 | 0.96 | 1.12 |
| ≥1grams | 3,625 | 3,137 | 1.18 | 1.20 | 1.24 | 1.12 | 1.06 | 1.81 | 1.09 | 1.04 | 1.15 |
| ≥5grams | 2,015 | 1,718 | 1.20 | 1.22 | 1.28 | 1.15 | 1.07 | 1.23 | 1.09 | 1.02 | 1.18 |
| ≥ 10grams | 1,133 | 946 | 1.23 | 1.12 | 1.34 | 1.18 | 1.07 | 1.30 | 1.11 | 1.00 | 1.22 |
| 1-4.9grams | 1,610 | 1,419 | 1.16 | 1.07 | 1.24 | 1.09 | 1.01 | 1.18 | 1.09 | 1.01 | 1.18 |
| 5-9.9grams | 882 | 772 | 1.16 | 1.06 | 1.28 | 1.11 | 1.00 | 1.23 | 1.08 | 0.97 | 1.20 |
| **By average daily dose & cumulative dose** | | | | | | | | | | | |
| DD <7.5 mg/day | | | | | | | | | | | |
| CD < 1gram | 1,040 | 969 | 1.09 | 1.00 | 1.19 | 1.05 | 0.95 | 1.15 | 1.06 | 0.96 | 1.16 |
| CD ≥1 grams | 2,121 | 1,816 | 1.19 | 1.12 | 1.27 | 1.12 | 1.05 | 1.20 | 1.11 | 1.03 | 1.19 |
| CD ≥5 grams | 1,105 | 912 | 1.24 | 1.13 | 1.35 | 1.19 | 1.08 | 1.31 | 1.14 | 1.04 | 1.26 |
| CD ≥10 grams | 569 | 464 | 1.26 | 1.11 | 1.42 | 1.24 | 1.09 | 1.42 | 1.17 | 1.03 | 1.34 |
| CD 1-4.9grams | 1,016 | 904 | 1.15 | 1.05 | 1.25 | 1.06 | 0.96 | 1.17 | 1.07 | 0.97 | 1.18 |
| CD 5-9.9 grams | 536 | 448 | 1.22 | 1.08 | 1.38 | 1.13 | 0.99 | 1.29 | 1.11 | 0.97 | 1.27 |
| DD 7.5-14.9 mg/day | | | | | | | | | | | |
| CD < 1 gram | 197 | 190 | 1.05 | 0.86 | 1.28 | 1.04 | 0.84 | 1.28 | 1.02 | 0.83 | 1.27 |
| CD ≥1 grams | 1,032 | 921 | 1.14 | 1.04 | 1.25 | 1.09 | 0.99 | 1.20 | 1.04 | 0.94 | 1.15 |
| CD ≥5 grams | 701 | 615 | 1.16 | 1.04 | 1.30 | 1.09 | 0.97 | 1.23 | 1.03 | 0.91 | 1.16 |
| CD ≥10 grams | 464 | 384 | 1.24 | 1.08 | 1.41 | 1.14 | 0.98 | 1.32 | 1.05 | 0.91 | 1.22 |
| CD 1-4.9grams | 331 | 306 | 1.10 | 0.94 | 1.28 | 1.08 | 0.91 | 1.27 | 1.07 | 0.90 | 1.26 |
| CD 5-9.9 grams | 237 | 231 | 1.04 | 0.87 | 1.25 | 1.02 | 0.84 | 1.24 | 0.98 | 0.81 | 1.19 |
| DD ≥15 mg/day | | | | | | | | | | | |
| CD < 1 gram | 203 | 197 | 1.04 | 0.86 | 1.27 | 0.97 | 0.79 | 1.19 | 0.97 | 0.78 | 1.19 |
| CD ≥1 grams | 472 | 400 | 1.20 | 1.05 | 1.37 | 1.18 | 1.02 | 1.36 | 1.16 | 1.00 | 1.34 |
| CD ≥5 grams | 209 | 191 | 1.12 | 0.92 | 1.36 | 1.12 | 0.91 | 1.39 | 1.08 | 0.87 | 1.34 |
| CD ≥10 grams | 100 | 98 | 1.04 | 0.79 | 1.37 | 1.05 | 0.78 | 1.42 | 0.99 | 0.73 | 1.34 |
| CD 1-4.9grams | 263 | 209 | 1.28 | 1.06 | 1.53 | 1.22 | 1.00 | 1.48 | 1.23 | 1.01 | 1.49 |
| CD 5-9.9 grams | 109 | 93 | 1.20 | 0.91 | 1.58 | 1.20 | 0.89 | 1.62 | 1.18 | 0.87 | 1.59 |
| (a) adjusted for: history of COPD, fracture, rheumatoid arthritis, inflammatory bowel disease, secondary osteoporosis, inhaled bronchodilators, antidepressants, anxiolytics and hypnotics, anticonvulsants | | | | | | | | | | | |
| (b) adjusted for confounds in model A plus bone medications (bisphosphonates, raloxifene, strontium, denosumab, calcium, vitamin D), calcitonin, and hormone replacement therapy | | | | | | | | | | | |
| Oral glucocorticoids exposure based on most recent glucocorticoid prescription prior to the index date: current (within 91-days), recent (92-182 days), past (183-364 days), and distant past (> 364 days). DD and CD were calculated among current users.  GC, Glucocorticoid; CD, Cumulative dose; DD, daily dose; OR, Odds ratio; CI, Confidence interval | | | | | | | | | | | |
